# Dangers of “Bonwill’s Method” of Anæsthesia

**Published:** 1881-02

**Authors:** 


### DANGERS OF "BONWILL'S METHOD" OF ANÆSTHESIA.

This method, as is well known, is by means of forced respirations. Dr. Henry Gibbons, of San Francisco, says of it, in the *Pacific Medical Journal*: —

"We suppose that most persons, most boys, at any rate, have experienced the sensation produced by blowing at a fire, substituting their lungs for a bellows. The proposed method is nothing more than an exaggeration of this. So far from regarding it as likely to prove useful, we look upon it as decidedly dangerous. It puts the brain in an abnormal condition by interfering with the circulation, and is almost equivalent to partial strangulation. We are surprised that any considerate physician should recommend such a violation of physiological laws."

